# Infection by the Parasite *Myxobolus bejeranoi* (Cnidaria: Myxozoa) Suppresses the Immune System of Hybrid Tilapia

**DOI:** 10.3390/microorganisms10101893

**Published:** 2022-09-23

**Authors:** Keren Maor-Landaw, Margarita Smirnov, Vera Brekhman, Maya Ofek-Lalzar, Tal Yahav, Tamar Lotan

**Affiliations:** 1 Marine Biology Department, The Leon H. Charney School of Marine Sciences, University of Haifa, Mt. Carmel, Haifa 3498838, Israel; 2 Central Fish Health Laboratory, Department of Fisheries and Aquaculture, Ministry of Agriculture and Rural Development, Nir David 10803, Israel; 3 Bioinformatic Unit, University of Haifa, Mt. Carmel, Haifa 3498838, Israel

**Keywords:** Myxozoa, Myxobolus, parasite, tilapia, infection, immune response, immune suppression, gills, head kidney, transcriptome

## Abstract

Myxozoa (Cnidaria) is a large group of microscopic obligate endoparasites that can cause emerging diseases, affecting wild fish populations and fisheries. Recently, the myxozoan *Myxobolus bejeranoi* was found to infect the gills of hybrid tilapia (Nile tilapia (*Oreochromis niloticus*) × Jordan/blue tilapia (*O. aureus*)), causing high morbidity and mortality. Here, we used comparative transcriptomics to elucidate the molecular processes occurring in the fish host following infection by *M. bejeranoi*. Fish were exposed to pond water containing actinospores for 24 h and the effects of minor, intermediate, and severe infections on the sporulation site, the gills, and on the hematopoietic organs, head kidney and spleen, were compared. Enrichment analysis for GO and KEGG pathways indicated immune system activation in gills at severe infection, whereas in the head kidney a broad immune suppression included deactivation of cytokines and GATA3 transcription factor responsible for T helper cell differentiation. In the spleen, the cytotoxic effector proteins perforin and granzyme B were downregulated and insulin, which may function as an immunomodulatory hormone inducing systemic immune suppression, was upregulated. These findings suggest that *M. bejeranoi* is a highly efficient parasite that disables the defense mechanisms of its fish host hybrid tilapia.

## 1. Introduction

The Tilapia (family Cichlidae), which is a recommended food item by the United Nations’ Food and Agriculture Organization, is the second most cultured fish worldwide [1], accounting for 60% of total production in Israel [2]. Tilapia have gained popularity in aquaculture due to their fast growth, tolerance to a wide range of environmental conditions, resistance to stress and ability to reproduce effectively in captivity [1]. Tilapia hybridization has been practiced in Israel since the early 1960s. The most popular hybrid in commercial use is an all-male *Oreochromis niloticus* (Nile tilapia) females × *O. aureus* (Jordan/Blue tilapia) males [3].

In the last 15 years, intense Myxozoa infections have been reported in Israeli fish ponds. Recently, the causative agent was classified as *Myxobolus bejeranoi*, which infects the hybrid tilapia at more than 50% prevalence [4]. The infection, which is limited to the gills and was not found in any other organ, can lead to impaired respiratory function and high mortality [4]. Therefore, *M. bejeranoi* infection of hybrid tilapia has high economic impact on commercial fish farms.

Myxozoa is a large group of microscopic obligate endoparasites affect wild and farmed fish populations, causing diseases such as whirling disease and proliferative kidney disease [5]. Recent morphological and phylogenomic analyses have placed Myxozoa within the phylum Cnidaria, which also contains corals, sea anemones, jellyfish, and hydroids (recently reviewed by [6]). Compared with their free-living cnidarian relatives, myxozoans have highly reduced body plans. Moreover, their genomes lack several key elements of signaling pathways and transcription factors that are hallmarks of multicellularity, but retain genes necessary for their function as obligate parasites [7,8]. The complex Myxozoan life cycle includes two hosts; a vertebrate, mostly fish, and an invertebrate, mostly worm [9,10]. Transmission between hosts is achieved by two distinct types of waterborne spores termed actinospores and myxospores [11,12].

Currently, there are no available treatments or vaccines against myxozoan diseases. Therefore, prevention measures or strategies that enhance the fish immune system have the most potential [13]. In recent years, knowledge of the immune system of fish and, particularly, of their immune response against parasites has greatly increased [14]. However, there are large gaps in our understanding of the molecular aspects of Myxozoa infection. Thus, elucidating the cellular processes that unfold in the fish host following infection is of great economic significance.

Teleost fish is the first taxonomic group to acquire both innate and adaptive immunity [13,15,16]. Teleost and mammalian immune systems share a repertoire of cells, including lymphocytes, monocytes, macrophages, granulocytes, thrombocytes, mast cells, non-specific cytotoxic cells, and possibly dendritic cells, as well as molecules such as T and B cell receptors, major histocompatibility complex (MHC), and immunoglobulins [17,18]. However, fish lack bone marrow and lymph nodes and their main lymphohematopoietic organs are the head kidney and spleen.

Typically, the first contact of myxozoans with the fish host is through mucosal surfaces, namely skin, gills, buccal cavity, or gastrointestinal tract [19]. Parasites can be eliminated by molecules present in the mucus, such as lysozyme, lectins, complement, and immunoglobulins; alternatively, they may pass undetected by the immune system [20,21]. At the following mucosal layer, the parasite will be challenged by various cell types such as macrophages, granulocytes, including mast cells, B cells, T cells and immunoglobulins [22]. After passing mucosal and epithelial barriers, the parasite travels through the bloodstream to its specific target tissue, where it proliferates. Some myxozoans are terminated in the blood by cellular and humoral immune factors [23,24,25]. Once the parasite is at the target tissue, the host activates immune mechanisms including immunoactivating and immunosuppressive cytokines [17,26,27]. A common histopathological response to myxozoan infection is the formation of granulomata, which encapsulate the parasite by connective and epithelioid tissue layers, thereby isolating it and preventing its dispersal to surrounding tissues [17,28]. However, some myxozoans can survive within this tissue and will eventually be able to release new spores to the environment (reviewed by [17]. The innate host response to myxozoan infection was previously described as a double-edge sword [13]. Whereas the absence of an immune response can result in coexistence or host death, hyperreaction can cause immune-related pathologies and will not necessarily stop parasite proliferation.

Here, we characterize the temporal progression of *M. bejeranoi* infection and the immune response of hybrid tilapia by performing transcriptomic analyses of both the sporulation site, the gills, and the immuno-organs head kidney and spleen. We present an interesting case study, where the myxozoan parasite displayed an immune evasion strategy of a thorough shutdown of the immune arsenal in head kidney. The consequence is an immune-deprived fish, which is expected to be highly susceptible to other opportunistic pathogens.

## 2. Materials and Methods

### 2.1. Evaluating the Infectious Potential of the Fish Pond Water

The study was conducted in an earthen fish pond with an area of 40,000 square meters at Reshafim Pisciculture, Beit She’an Valley, Israel. Juvenile hybrid tilapia fish (Nile tilapia (*Oreochromis niloticus*) × blue tilapia (*O. aureus*)) were translocated into the pond on 12 July 2020 for commercial use. The hybrids genetic diversity is unknown since mix of hybrids have been bred by the local aquaculture industry for many generations. The level of infectious actinospores in the pond was monitored throughout the spring and summer of 2020 by screening for actinospores in the water and by assessing the percentage of infected fish in the pond. Water temperatures were recorded constantly using a temperature data logger (HOBO).

### 2.2. Detection of Waterborne Actinospores, Filtering and DNA Extraction

Water samples taken from the pond during early spring 2020 were filtered first through a 60-µm pore-size nitrocellulose membrane (Merck Millipore Ltd., Ref: NY1104700) and then through an 11-µm membrane. The first membrane filtered out much of the larger plankton and detritus, whereas the pore size of the second membrane was appropriate for trapping *M. bejeranoi* actinospores. The 11-µm membrane was collected after filtering 1–2 L of water, as one replicate, and was stored at −20 °C for DNA extraction.

Next, filter membrane was incubated with 1 mL of lysis buffer (40 mM EDTA, 50 mM Tris HCl (pH 8.3) and 0.75 M sucrose), 10 mg/mL of proteinase K, and 10% SDS, for 1 h at 55 °C with occasional vortexing, as previously described [29], with minor modifications. Following phenol-chloroform-isoamyl alcohol phase separation, 1/10 volume of 3 M sodium acetate (NaOAc; pH 5.2) was added along with 2 volumes of EtOH. Following incubation for 20 min at −80 °C and 15 min centrifugation at 4 °C, DNA pellet was washed with 80% EtOH. DNA quantification and purity were assessed using Nanodrop 2000c spectrophotometer (ThermoScientific, Waltham, MA, USA).

### 2.3. PCR Analysis

From the obtained DNA samples, *M. bejeranoi* small subunit ribosomal RNA gene (SSU rDNA; NCBI accession number MF401455) [4] was amplified. Universal eukaryotic primers for 18S [30] were utilized as a positive control (Supplementary Table S1). PCR was performed in 25 μL volumes with 0.02 unit/μL of Phusion Hot Start Flex DNA Polymerase (New England BioLabs, Ipswich, MA, USA), 1× of Phusion HF 5× Buffer, 200 μM of dNTPs, 0.5 μM of forward and reverse primers, and 1 μL (10 ng) of template. Denaturation of DNA (98 °C for 5 min) was followed by 35 cycles of amplification (98 °C for 10 s, 67 °C for 10 s, and 72 °C for 30 s), ending with a 5 min extension (72 °C). PCR products were run in a 1% agarose gel and the presence of actinospores was confirmed by a positive result for *M. bejeranoi* primers.

### 2.4. Evaluating Percentage of Infected Fish

Hybrid tilapia fish that hatched on 27 May 2020 were introduced to the pond using confined cages of ~100 L (100 cm × 30 cm × 30 cm) for a limited exposure time of either one week or one day. The cages were tailor-made of a PVC frame coated with a 5 mm sized mesh, which allowed free flow of water from the pond. Additionally, to assess the effect of a continuous exposure to the infectious actinospores in the pond water, fish were sampled from outside the cage. Fish were euthanized with 1 mL/L of 2-phenoxyethanol (Sigma-Aldrich, St. Louis, MO, USA) and gills (*n* = 10−30) were sampled into TNES-urea buffer (pH 8.0) containing 10 mM Tris-HCl, 125 mM NaCl, 10 mM EDTA (pH 8.0), 0.5% SDS and 4 M urea.

Gill tissue was digested in TNES-urea buffer supplemented with 100 μg/mL proteinase K (Biological Industries), following a previously described protocol [31]. PCR was conducted as described above, but with 100 ng of extracted DNA template.

### 2.5. Transcriptome Experimental Design

On 1 September 2020, 400 hybrid tilapia fish with a mean weight of 4.98 g were introduced to the pond using four confined cages of ~100 L (100 cm × 30 cm × 30 cm). The mean water temperature during the experiment was 30.3 °C. Before the experiment, five representative fish were subjected to thorough parasitological examination, which showed no indication for their presence. Additionally, plating of spleen and kidney samples on blood tryptic soy agar (TSA; Novomed, Jerusalem, Israel), brain heart infusion agar (BHI; Oxoid, UK) and thiosulfate citrate bile salts sucrose (TCBS; Himedia, Mumbai, India) resulted in no microbiological growth. After a 24 h exposure, the fish were randomly translocated to four 100-L indoor aquarium tanks at the Central Fish Health Laboratory, Nir David. Tanks had a flow-through system with dechlorinated tap water at a temperature of ~25 °C. Fish were fed daily with commercial fish pellets.

**Figure 1:**
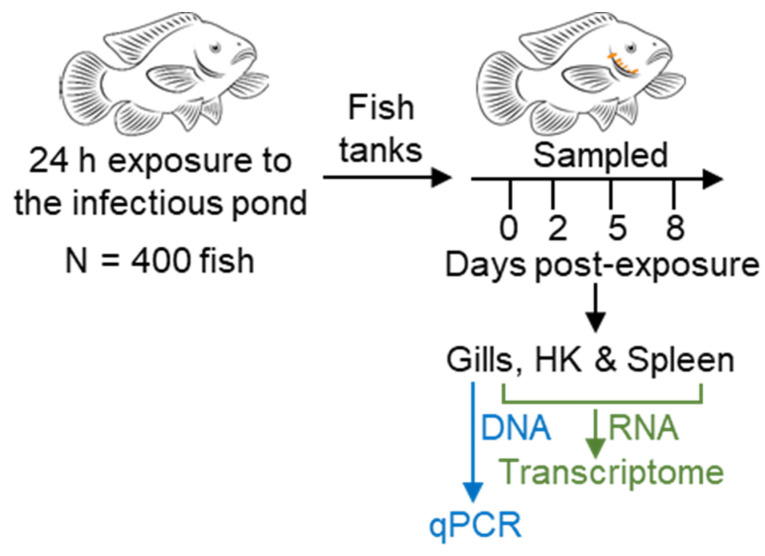
Experimental design. Approximately 400 3-month-old hybrid tilapia fish (Nile tilapia (*Oreochromis niloticus*) × blue tilapia (*O. aureus*)) were introduced to an earthen pond at Reshafim Pisciculture, Beit She’an Valley, Israel, in four confined cages. After 24 h of exposure to the pond water, fish were translocated to 100-L indoor aquarium tanks in the Central Fish Health Laboratory, Nir David. Fish were sampled immediately and after 2, 5, and 8 days. To study *M. bejeranoi* infection intensity, DNA samples were extracted from gill tissue and to analyze host response, RNA samples were extracted from gill tissue and the immuno-organs HK and spleen.

Fish were sampled (*n* = 10–30) immediately (time point 0) and at 2, 5, and 8 days post-exposure. At each time point, fish were euthanized (1 mL/L 2-phenoxyethanol) and whole-gill tissue (four and a half gill lamellae) from one side, head kidney (HK) and spleen were collected and snap-frozen in liquid nitrogen (Figure 1). Each sampling point included fish from all four tanks in order to avoid tank effects.

### 2.6. RNA and DNA Extraction

Simultaneous extraction of RNA and DNA from gill tissue and RNA extraction from internal organs were performed using TRIzol Reagent (Themo Scientific) according to the manufacturer’s instructions, with minor modifications. For each 0.1 g of frozen sample, 1.5 mL of TRIzol was added and the mixture was homogenized using four 3 mm glass beads (CS Chemicals Ltd., Ahmedabad, India) and a TissueLyser II (Qiagen, Hilden, Germany) for 3 min at 30 Hz. Following incubation with chloroform (Sigma-Aldrich, St. Louis, MO, USA) and centrifugation of 15 min at 12,000× *g* and 4 °C, the upper aqueous phase containing RNA was retrieved, while the interphase and organic phases were utilized for DNA extraction. Extracted RNA was treated in-column with DNase I (Ambion, Austin, TX, USA) according to RNA Clean & Concentrator-25 kit (Zymo Research, Irvine, CA, USA). The concentration of RNA and DNA was measured using a NanoDrop 2000c spectrophotometer (ThermoScientific, Waltham, MA, USA), and RNA integrity (RIN > 7; mean, 8.5) was assessed by a 2200 TapeStation System (Agilent Technologies, Santa Clara, CA, USA).

### 2.7. Evaluation of Infection Severity by Quantitative RT-PCR

To evaluate *M. bejeranoi* infection progression in the fish gills, quantitative PCR (RT-PCR) was applied to DNA extracted from gills. Primers were designed to amplify *M. bejeranoi* small subunit ribosomal RNA gene (SSU rDNA) (NCBI accession number MF401455) [4] (Supplementary Table S1). Tilapia β-actin gene was used as a normalizer [32] for the comparative ΔΔCTs method. RT-PCR was performed using Step One Plus (Applied Bio systems) 96-well machine. A DNA sample of 25 ng was used in technical triplicates for 10 µL RT-PCR reactions including 5 µL of Fast SYBR Green Master Mix (Applied Bio systems) and 0.5 µL each of forward and reverse primer, for 40 cycles. To test for nonspecific amplification, a melt curve obtained by incubating the reactions for 15 s at 0.3 °C increments between 60 °C and 90 °C was generated for each amplicon. Primer efficiencies for *M. bejeranoi* SSU rDNA and tilapia β-actin were determined using a standard curve analysis with a 5-fold dilution series, and were calculated to be 98.6% and 93.3%, respectively.

The computed RT-PCR relative quantity (RQ) was denoted as the relative infection severity index, which was categorized into three infection stages: minor (stage 1; RQ, 0.1–1; mean, ~0.6), intermediate (stage 2; RQ, 32–57; mean, ~45), and severe (stage 3; RQ, 110–300; mean, ~212). Five replicates from each category from gill tissue, spleen, and HK (45 RNA samples in total) were sent for sequencing.

### 2.8. Library Preparation and Sequencing

RNA measurements, library preparation and sequencing were performed at the Technion Genome Center, Technion-Israel Institute of Technology, Haifa, Israel. RNA concentration was measured using Qubit 4 Fluorometer (ThermoFisher Scientific) and RNA quality was measured using Agilent 2200 TapeStation (Agilent). RNA sequencing libraries were prepared using the CEL-Seq2 protocol, as described by Hashimshony et al. [33], with one modification of using 2 ng purified RNA instead of single cells as input for library preparation. The CEL-Seq2 libraries were analyzed for average fragment size using Agilent 2200 TapeStation and concentration was measured using Qubit 4 Fluorometer (ThermoFisher Scientific). The libraries were sequenced on the Illumina NextSeq 2000 sequencer (Illumina), 12 bases for read 1 and 65 bases for read 2. Demultiplexing was performed in two steps. First, Illumina demultiplexing was performed using bcl2fastq Illumina software with the following parameters: barcode-mismatches = 1, minimum-trimmed-read-length = 0, and mask-short-adapter-reads = 0. Second, Cell-seq demultiplexing using the published pipeline [33] was executed with the following parameters: min_bc_quality = 10, bc_length = 6, umi_length = 6, and cut_length = 70.

### 2.9. Quantification of Gene Expression and Enrichment Analysis

Raw sequence reads were filtered and trimmed for quality using *Cutadapt* (v3.4) [34]. Filtered reads were mapped to *O. niloticus* genome (Ensembl release 104; http://ftp.ensembl.org/pub/release-104/fasta/oreochromis_niloticus/) (accessed on 3 October 2021) and to the *O. aureus* genome (Ensembl release 104; http://ftp.ensembl.org/pub/release-104/fasta/oreochromis_aureus/) (accessed on 3 October 2021). Reads were mapped to the genome using STAR (v2.7.7a) [35] with standard parameters and the annotation file for optimization of mapping. Mapping percentages were between 86.2% and 92.3% for *O. niloticus* and between 74.9% and 87.3% for *O. aureus* (mean difference of 7.5% between fish species). Due to improved mapping to the *O. niloticus* genome, we continued to quantification and analysis using this genome. The HTseq tool [36] (https://github.com/yanailab/CEL-Seq-pipeline/blob/stable/htseq_wrapper.py) (accessed on 22 September 2021) was used for the construction of raw read-count matrices for each of the samples. DESeq2 [37] with default settings was used for identification of differentially expressed genes and determination of their log-fold change between different levels on infection (infection levels 2 vs. 1; 3 vs. 1) for each tissue. For differentially expressed genes, adjusted *p*-value < 0.1 was considered statistically significant. For gene set enrichment analysis (GSEA) [38], a pre-ranked list of genes was generated based on log-fold change. Using the ‘fgsea’ package in R  (v.1.18.0) [39], GSEA was performed with Gene ontology (GO) and Kyoto encyclopedia of genes and genomes (KEGG) gene set annotations. Minimum and maximum gene set sizes were set to 10 and 1000, respectively, and 1000 permutations were performed.

A process with a positive or negative normalized enrichment score (NES) was denoted as up-or down-regulated, respectively. Significantly enriched gene sets were filtered based on a cutoff of q  <  0.25 [38] and were clustered based on shared genes. For this purpose, pair-wise Jaccard distances were calculated between each pair of enriched GO or KEGG terms based on binary table indicating the presence or absence of each gene in each term. Jaccard distances were calculated using function ‘vegdist’ in R package ‘vegan’ (v2.5.7) (https://cran.r-project.org/web/packages/vegan/vegan.pdf, accessed on 27 October 2021). The Jaccard similarity matrix was then used for generation of a similarity network among terms, which was calculated and visualized with Cytoscape (v3.9.0).

An enrichment score was calculated for each group of GO/KEGG terms, which was defined as the minus log of the geometric mean of the all the *p*-values of the GO categories within the group [40]. Heat maps were generated using shinyheatmap (http://shinyheatmap.com) (accessed on 11 April 2022) using the default parameters [41]. Networks of highly interconnected proteins were generated using the STRING 11.5 database [42] and exported to Cytoscape [43] for graphical editing.

## 3. Results

**Figure 2:**
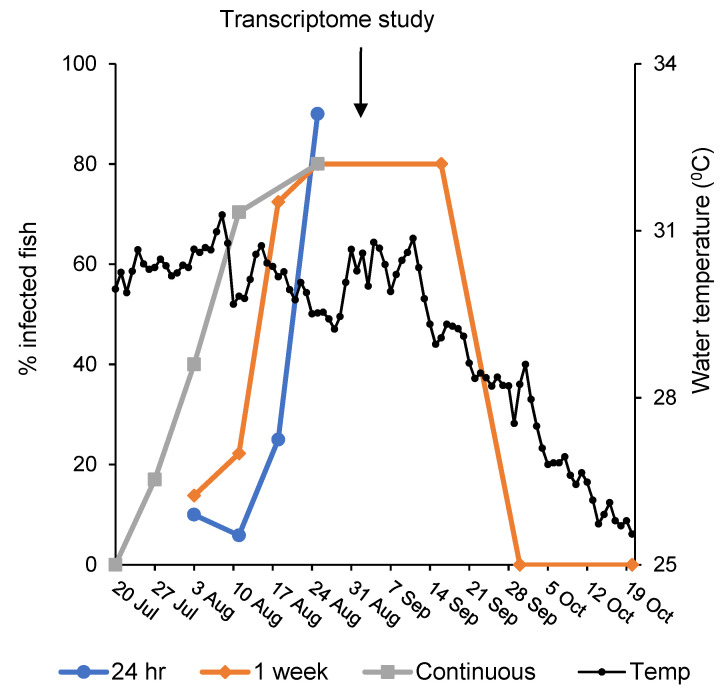
Infectious season of the pond water through 2020. Percentages of infected fish following a continuous exposure (grey line) or limited exposures of 24 h (blue line) or one week (orange line) to the pond water (*n* = 10–30) throughout the summer of 2020. Water temperatures (black line) were recorded using a data logger placed at a depth of 2 m in the pond water.

Before conducting the transcriptomic analysis, the infectious potential of the actinospores in the fish pond water was evaluated. The first indication of the presence of actinospores in the pond water was found on 27 July 2020, when the extracted DNA from filtered membrane was PCR-positive for myxozoan actinospores. In addition, 17% of tested fish were positive for M. bejeranoi infection (Figure 2). The numbers of infected fish increased gradually, and the peak was at late August–mid-September. On 25 August, 90% of the fish were infected after an exposure of only 24 h to the pond water. By the end of September, the pond water no longer contained actinospores, consistent with the decline in water temperatures (Figure 2).

To investigate the molecular processes involved in the progression of *M. bejeranoi* infection in cultured hybrid tilapia, we performed a transcriptomic study on fish collected when infection rates were at their peak. All sampled fish were exposed to the same natural conditions of the pond for 24 h. We analyzed tissues from the sporulation site, the gills, and from the hematopoietic organs HK and spleen. Additionally, we compared the obtained data between fish with minor, intermediate and severe infection levels.

**Figure 3:**
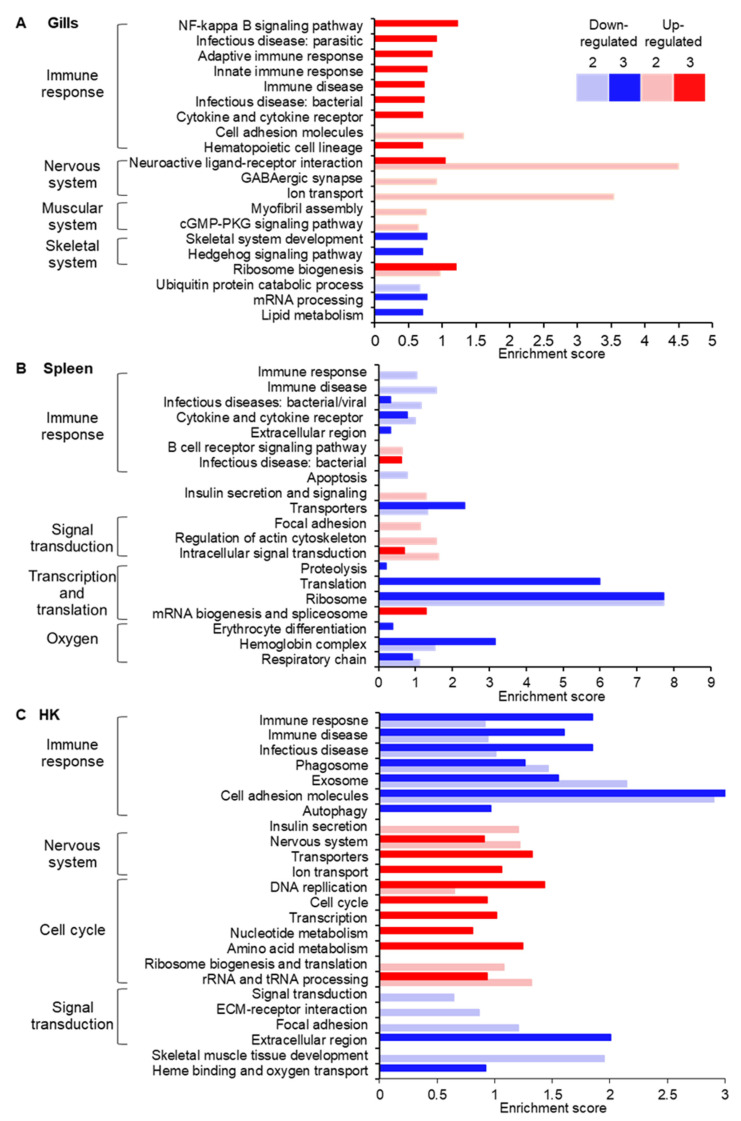
Gene ontology and KEGG pathways enrichment analysis. Enrichment scores, calculated from adjusted *p*-values, are presented for gills (**A**), spleen (**B**), and HK (**C**) at intermediate (2) (lighter shades) and severe (3) (darker shades) infection stages. Up- and down-regulated processes are indicated by red and blue colors, respectively. Clusters of cellular processes are marked. See Supplementary Table S2 for additional details of GO and KEGG terms.

Enrichment analysis for GO and KEGG pathways revealed distinct cellular patterns for each examined tissue, with some similarities between the two hematopoietic organs (Figure 3, Supplementary Table S2). In the sporulation site, the nervous system (GABAergic synapse pathway) and muscular system (myofibril assembly and cGMP-PKD signaling pathway) were up-regulated already at the intermediate infection level (stage 2). However, processes related to the innate and adaptive immune response, such as NF-kappa B signaling pathway, protein interaction with cytokine and cytokine receptor, immune disease, and infectious disease, were activated only upon severe infection (stage 3). Ribosome biogenesis was up-regulated at both these stages. Among the down-regulated categories were skeletal system development, hedgehog signaling pathway, and lipid metabolism (Figure 3A).

In the spleen, while some immune system categories, i.e., infectious diseases: bacterial/viral, immune disease, protein interaction with cytokine and cytokine receptor were down-regulated, B cell receptor signaling pathway and infectious disease: bacterial were up-regulated at stages 2 and 3, respectively. Other processes that were significantly enriched included down-regulation of apoptosis and ribosome and translation, and up-regulation of signal transduction (Figure 3B). In both spleen and HK, enrichment analysis revealed processes involved in oxygen maintenance in the cell, namely heme binding, oxygen transport, and respiratory chain and erythrocyte differentiation (Figure 3B,C). At intermediate infection, insulin secretion was activated in the HK and spleen and in the latter, additional insulin-related processes such as insulin signaling pathway and insulin resistance were up-regulated.

Results of the HK analysis showed a different trend regarding the immune system. Immune response processes such as immune disease, infectious disease, Th1 and Th2 cell differentiation, antigen processing and presentation, cytokine signaling, and natural killer mediated cytotoxicity were down-regulated (Figure 3C). This trend started at stage 2 of the infection and intensified at stage 3, as additional enriched processes and higher enrichment scores were observed. As in the gills, in the HK the nervous system was activated upon the progression of the infection. Whereas DNA replication, rRNA and tRNA processing and translation increased already at stage 2, at stage 3 more processes related to cell cycle and replication and the required substrates, such as nucleotide and amino acid metabolism, were up-regulated. Several categories related to signal transduction and skeletal muscle tissue development were down-regulated.

**Figure 4:**
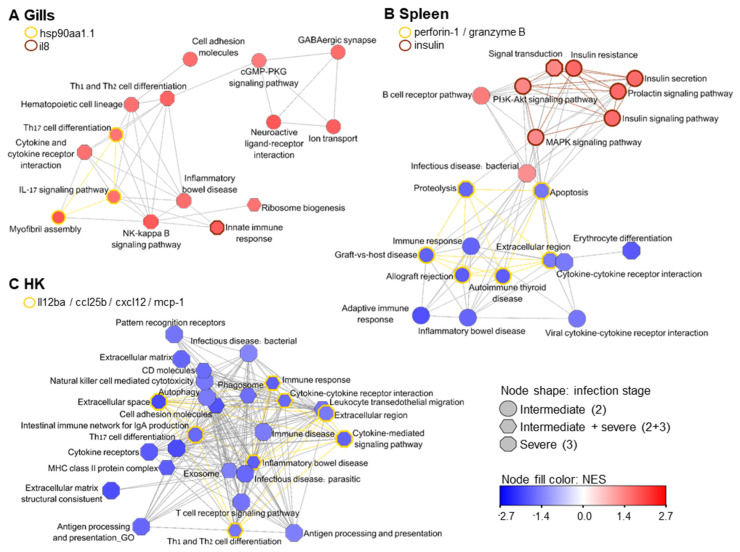
Network analysis for enriched processes (GO/KEGG pathways) based on shared genes. (**A**) Enriched processes in the gills were clustered according to the state of the infection. Intermediate infection are enriched with nervous and muscular-related terms that are linked to severe infection showing enrichment of a cluster of immune-related terms. (**B**) Enriched processes that are related to immune function or signaling are presented for the spleen. (**C**) Immune-related processes in HK. Node fill is color-scaled according to mean NES (up-regulated, red; down-regulated, blue), and node shape indicates the infectious stage (see legend). Edge lengths were derived from shared genes between the connected nodes. Border-colored nodes and colored edges (yellow/brown) represent a differentially expressed signaling/effector gene that participates in the process, as each legend details. See Supplementary Table S3 for additional details of the terms.

Figure 4 shows for each tissue networks of enriched GO processes and KEGG pathways based on the shared genes between them (Supplementary Table S3). In the gills (Figure 4A), GABAergic synapse and neuroactive ligand-receptor interaction at stage 2 are connected to stage 3 Th1 and Th2 cell differentiation of the immune cluster via cGMP-PKD signaling pathway. Moreover, the process of myofibril assembly, which relates to muscle activity and was up-regulated at stage 2, is linked to IL-17 signaling pathway and Th17 cell differentiation, which were enriched at severe infection. The shared gene that governs these processes was heat shock protein 90 alpha (hsp90aa1.1) (Supplementary Table S4), with log2 fold change (log2FC) of 3.2 and 3.9 at infection stages 2 and 3, respectively. A secreted signaling molecule with chemokine activity that participated in the innate immune response process was an uncharacterized protein with interleukin-8-like domains. This gene )LOC100699978 (was up-regulated in all examined tissues at the severe infection level.

In the spleen, three contigs representing the effector protein perforin-1, which is responsible for cytolysis, with membrane attack domain, as well as the serine protease granzyme B, were down-regulated (Figure 4B, Supplementary Table S4). These genes are involved in proteolysis, apoptosis, and autoimmune diseases, which were also down-regulated. The cluster of up-regulated processes contains two immune-related pathways, as well as signaling pathways that are related to insulin secretion.

Figure 4C shows deactivated immune-related GO and KEGG pathways in HK, which were clustered based on shared gene expression. Down-regulation of extracellular cytokine activity (processes Th1 and Th2 cell differentiation, inflammatory bowel disease, cytokine-mediated signaling pathway, and immune response) was accompanied by expression of interleukin-12 subunit beta (*il12ba*), chemokine (C-C motif) ligand 25b (*ccl25b*), chemokine (C-X-C motif) ligand 12a (stromal cell-derived factor 1; *cxcl12*), and monocyte chemotactic protein 1B (*mcp-1*) (Supplementary Table S4).

**Figure 5:**
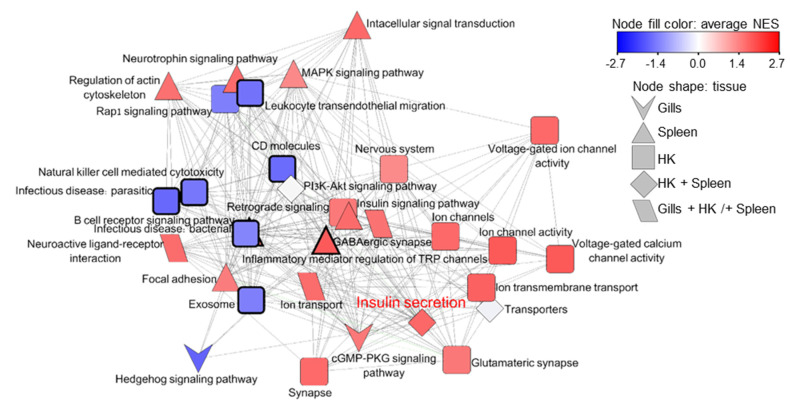
Insulin secretion network analysis for enriched GO and KEGG pathways. Enriched processes from gills, spleen, and HK were organized into a network based on shared genes. Edge lengths are derived from common genes between the connected nodes. Network presents processes that are first neighbors to insulin secretion (labeled in red). Node fill is color-scaled according to mean NES (up-regulated in red, down-regulated in blue), and node shape indicates the tissue, as legend details. Processes that are related to the immune system are with thicker node border. See Supplementary Table S5 for additional details of the terms.

The pathway of insulin secretion was up-regulated in both hematopoietic organs at intermediate infection and insulin gene was significantly up-regulated in the spleen with an extremely high fold change of ~560 (Supplementary Table S4). This gene participates in signal transduction process, which was enriched in the gills at stage 3. Therefore, we analyzed the network of insulin first neighbors based on shared genes among all enriched processes in the three tested organs (Figure 5, Supplementary Table S5). Insulin secretion was related to signaling pathways, such as MAPK, PI3-AKT, and cGMP-PKG; to the nervous system; and to the immune processes natural killer cell mediated cytotoxicity, CD molecules, exosome, leukocyte transendothelial migration, and infectious diseases, which are all down-regulated, with the exception of the up-regulated processes B cell receptor signaling pathway and inflammatory mediator regulation of TRP channels.

**Figure 6:**
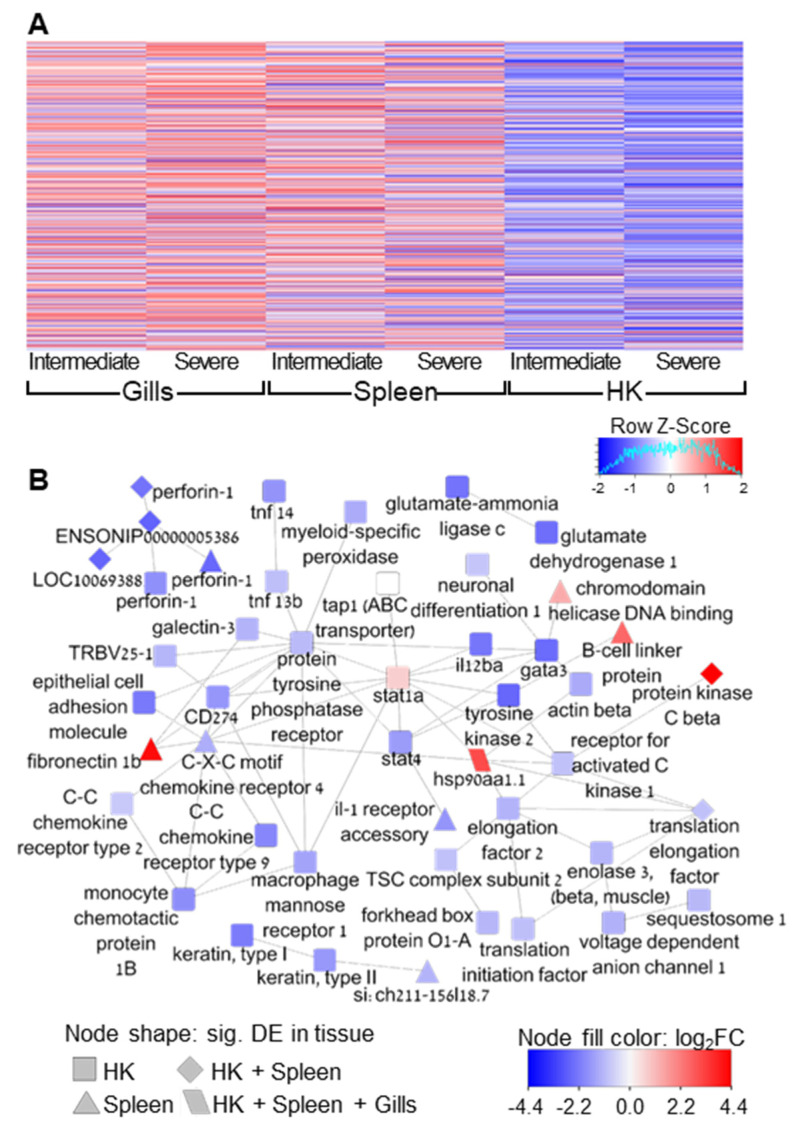
Expression patterns of immune-related genes and network analysis. (**A**) Heat map showing hierarchical clustering of gene expression patterns (log2FC) in the three tissues at intermediate and severe infection stages (up-regulated, red; down-regulated, blue). (**B**) Network of significantly differentially expressed genes based on protein interactions (STRING database). Node fill is color-scaled according to log2 FC (up-regulated, red; down-regulated, blue) and node shape indicates the tissue, as the legend details. See Supplementary Table S6 for additional details of the genes labels.

The gene expression patterns of all immune-related genes, which are shown in a hierarchical clustering heat map (Figure 6A), illustrate the differences in tissue response to infection. The sporulation site was characterized by up-regulation, HK mostly by down-regulation, while the spleen was in-between them, displaying a mixed effect in the expression of the immune-related genes.

STRING interaction network of differentially expressed immune-related genes was found to be significantly enriched with protein–protein interactions (*p* = 1.78 × 10^−15^) (Figure 6B, Supplementary Table S6). The mean number of interactions per protein (mean node degree) was 2.2. However, some key proteins had multiple interactions, such as signal transduction and activator of transcription 1 (stat1a), which had 11 first neighbors. This gene was up-regulated in HK at stage 3 (*p* = 0.01), but the adjusted *p*-value was borderline insignificant (0.13). We did not discriminate it from the network due to its multiple interconnections. Another transcription factor that had numerous interactions was GATA binding protein 3 (gata3), which was down-regulated in HK. Protein tyrosine phosphatase receptor type C showed a similar trend; however, it had multiple connections (12) and it seemed central in the network. Finally, HSP90 was uniquely found to be significantly up-regulated in all tissues and all infection stages and had 6 protein interactions, for example with elongation factors, stat1, and chemokine receptor.

## 4. Discussion

The most important function of a parasite is to secure its transmission to a new host by surviving the host immune response [44,45]. Therefore, many parasites have developed varied strategies to avoid detection, suppress immunity and deviate immune attack mechanisms [46]. Our results imply that the myxozoan parasite *M. bejeranoi* triggers a systemic immune suppression in its host, the fish hybrid tilapia. Although we documented an activation of the immune system at the local sporulation site, the gills, this occurred only at a severe infection stage. Moreover, the hematopoietic organs responsible for systemic immunity seem to fail in the combat against the infection already at intermediate infection level. Immune suppression is well known in parasitic infection. However, the literature on fish-myxozoan interaction is ambiguous, as some studies indicate immune activation [47,48] while others report suppression [49,50,51,52]. Noteworthy, these studies differ in the studied Myxozoa-host species, target tissue, and experimental layout, including the stage of the infection.

Prolongation of host cell life through modulation of apoptosis is another tactic employed by parasites to ensure their proliferation ([46,53] and in Myxozoa [54]). The down-regulation of apoptosis in spleen may further indicate impaired immune response, whereas the increased cell maintenance and cell cycle processes in HK may reflect efforts to sustain host cell life.

**Figure 7:**
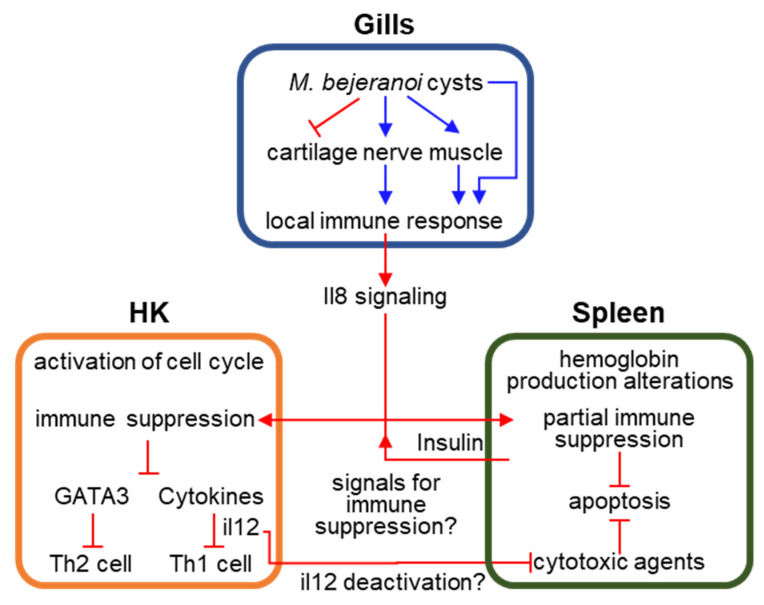
A Model illustrating the key findings and open questions regarding *Myxobolus bejeranoi* infection of hybrid tilapia. *M. bejeranoi* sporulation takes place in the gills within encapsulated cysts. The cysts, which are located on striated muscles at the base of the gill filament, trigger modifications in local nerve, muscle, and cartilage. As infection severity increases, the immune system is activated. We hypothesize that a signal is sent from the gills to the hematopoietic organs HK and spleen, leading to a systemic immune suppression. While a cytokine with domains of interleukin 8 (il8) was up-regulated in all examined tissues, the fundamental question regarding the nature of this signal remains unanswered. In HK, cell cycle processes are activated, while the immune system is suppressed. Suppression includes deactivation of the master regulator transcription factor *GATA3*, which leads to less differentiation of Th2 cells and deactivation of cytokines. Amongst the latter is interleukin 12 (il12), whose inactivation leads to decrease in Th2 cells differentiation and may induce down-regulation of cytotoxic agents (perforin, granzyme B) in natural killer cells in the spleen. Thus, deactivation of these cytotoxic agents may lead to decrease in apoptosis. Spleen exhibited alterations in the erythrocyte machinery and hemoglobin production. Insulin levels were highly increased in the spleen, suggesting that this immunomodulatory hormone may further signal for immune suppression.

We hypothesize that the signal for the systemic immune suppression in HK and spleen originates in the site of sporulation. However, the fundamental question of the nature of this signal remains unanswered. We mined our data for differentially expressed genes encoding for signaling molecules that could be secreted by gill cells and reach the HK through the blood stream via the branchial efferent arteries. We found that *LOC100699978* is up-regulated in the gills at stage 3, and also in spleen and HK. It contains a domain of chemokine interleukin-8-like, which is a pro-inflammatory cytokine that was shown in fish to have a chemotactic effect on HK leukocytes and macrophages [55,56,57]. Another gene-of-interest is insulin, which was up-regulated at intermediate infection in spleen with extremely high fold change of ~560. Insulin is an important signaling molecule regulating a wide array of processes in fish [58]. Moreover, studies indicate that insulin could be an immunomodulatory hormone in fish, inducing immune suppression [59,60,61]. The high involvement of insulin secretion in down-regulated immune processes in HK and spleen further supports its possible role as the signal for immune shutdown in tilapia following *M. bejeranoi* infection. However, this intriguing hypothesis should be further examined in future studies (Figure 7).

A substantial immune response in the gills commenced only at severe infection stage. However, the gill tissue clearly responds to the infection. As cysts are located on striated muscle of the gill filament base [4], they press on local nerves and muscle tissue, which is expected to cause up-regulation of related genes. Indeed, ribosome biogenesis, which is required to satisfy the increased demand for gene activation, was up-regulated. Further, changes in gill branchial arches cartilage may be indicated by the down-regulation in skeletal system development and Hedgehog signaling pathway. Formation of cartilage has been reported to be tightly regulated by Hedgehog signaling [62,63], with indications of a similar function also in fish chondrocytes, which are responsible for cartilage formation [64,65,66,67].

In teleost fish, HK and spleen are the two main lymphoid and hematopoietic organs. [68]. Therefore, it is unsurprising that we found some similarities in HK and spleen differentially expressed processes. Gene expression patterns common to HK and spleen were previously reported in turbot severely infected with the myxozoan *Enteromyxum scophthalmi* [54]. Amongst the shared processes in our study are immune-related processes, insulin secretion, and heme binding. The latter, along with down-regulation of respiratory chain and erythrocyte differentiation in the spleen, may indicate alterations in the erythrocyte machinery and hemoglobin production [54]. Interestingly, the spleen displayed an immune response that was halfway between the activated gills and suppressed HK. Some pathogens operate by disrupting the host cytoskeleton [46], which correlates with the findings of modification of cytoskeleton-related genes in enteromyxosis [54], enteronecrosis [52], proliferative kidney disease [47], and the activation in the spleen in this study.

The immune system cluster in HK includes down-regulated exosomes and autophagy. Exosomes play an important role in antigen presentation, inflammation and pathogenesis [69,70] and the autophagy interface is essential for immunity and inflammation [71]. In fish, it was found that HK cells secrete exosomes when the immune system is triggered by a viral infection [72,73]. Inhibition of autophagy was detected in a transcriptomic study of a sea bream infected by a flatworm [74], and in rainbow trout infected with the myxozoan parasite *Tetracapsuloides bryosalmonae* [75]. The involvement of both processes in fish immune suppression following Myxozoa infection is still unclear.

Differentially expressed cytokines drive inflammatory signals to regulate the capacity of phagocytes to destroy the invading pathogen. The profile and magnitude of cytokine response determine whether the immune responses will be beneficial or detrimental to the host [76]. The four down-regulated cytokines we identified in HK at severe infection were previously found to have prominent roles in fish immune system. IL-12 induced Th1-type immunity [77] and was down-regulated following a parasitic infection [78] or a LPS-induced inflammation [79]. CCL25 promoted leukocytes and macrophages recruitment [80,81,82] and was activated in carp’s liver infected with *Myxobolus wulii*. However, the opposite trend was observed in fish infected with helminth [83], ciliated parasite [84], virus [85], or bacteria [86]. In mammals, CXCL12 affects the migration, proliferation and differentiation of leukocytes [87]. Correspondingly, in myxozoan-infected fish, CXCL12 was up-regulated [47,88], but following viral infection the gene was down-regulated [85]. MCP-1 (also termed CCL2) has a positive effect on the chemotaxis of monocytes/macrophages and neutrophils [89] and, thus, was activated in a bacterial [89] or viral infection [90]. While several of the above-cited studies aimed at elucidating the function of these cytokines in fish, this field of research is in its infancy and the reported trends in gene expression upon exposure to various pathogens are often inconsistent, thought in some cases consensus of the immune modulation was observed (PKD, enteromyxosis). In our study, all of these signaling molecules were significantly down-regulated along with the corresponding GO/KEGG pathways. Nevertheless, the question of what induced their transcriptional down-regulation in *M. bejeranoi*-infected hybrid tilapia remains to be answered.

The common dogma is that the balance between Th1 and Th2 cell types determines the susceptibility to disease states [91]. Our results indicate deactivation of both paths. In fish and mammals, Th1 cells orchestrate immune response to intracellular pathogens by activating macrophages and cytotoxic T cells [13]. The differentiation process to Th1 is promoted by (amongst others) IL-12 and controlled by the transcription factor STAT1 [92]. IL-12 is a potent activator of natural killer (NK) cell-mediated cytotoxicity, which activates transcription of perforin and granzyme [93]. The de-activation of IL-12 in HK might have triggered deactivation of these effector cytotoxic agents in the spleen. In mammals, cytotoxic T cells kill target cells via the secretory pathway that is governed by the cytotoxins perforin, granzyme, and granulysin/NK-lysin. When CD8^+^ T cells recognize peptides presented by MHC-I molecules, perforin is released, forming pores in the membrane of target cell. Then, granzyme B enters the cytoplasm through these pores and induces apoptosis [92]. In fish, non-specific cytotoxic cell activity in HK was higher following infection by the myxozoan *Enteromyxum leei* [26]. Nonetheless, while some studies support this result and show higher expression of perforin, granzyme, or NK-lysin in fish infected by *E. leei* [91], *Ceratonova shasta* [25,52] or *Myxobolus honghuensis* [88], other works in fish infected by *E. scophthalmi* [54] or *C. shasta* [52] report down-regulation.

Th2 cells are related to immune response to extracellular parasites and promote B cell proliferation and antibody production [94]. In our study, indication to the suppression of this path is in the down-regulation of the master regulator GATA3 transcription factor, which drives Th2 cell differentiation [94] (Figure 7). GATA3 was previously found to be down-regulated in fish spleen and HK following exposure to myxozoan parasites *E. leei* and *T. bryosalmonae* [91,95]; however, in other reports it was up-regulated [25,51,88,96].

In the case study of *M. bejeranoi* vs. hybrid tilapia, the myxozoan parasite appears to be highly efficient in silencing the immune response of the host, prolonging host cell life and proliferating without much interruption. Thus, as most of the fish defense mechanisms are down, the parasite renders its host more susceptible to other opportunistic pathogens. Aquaculture earthen ponds are home to highly dynamic microbial communities [97]. These include *Aeromonas* [98], the causative agent of motile Aeromonas septicaemia disease, and the highly contagious tilapia lake virus (TiLV) [99]. Coinfection of *M. bejeranoi* and another pathogen [4] is expected be detrimental to tilapia health condition and annual fish stocks, as heterogeneous coinfection studies in other myxozoan species were shown to have synergistic affects [100,101]. Elucidating gene expression patterns in the myxozoan during infection will improve the understanding of the processes enabling the highly compact myxozoan parasite to effectively immune-suppress its host for its own needs.

## 5. Conclusions

This study examined how infection with the parasitic myxozoan *M. bejeranoi* affects cellular processes and immune response of hybrid tilapia, cultured in its natural farming environment. Analysis of the sporulation site indicated that the nervous and muscular systems were triggered along with the immune system. However, a systemic immune suppression was documented mostly at the primary hematopoietic organ, the head kidney, which included significant down-regulation of cytokines and transcription factors. The magnitude of the shutdown was manifested also in the deactivation of cytotoxic effectors in the spleen. For a parasite such as *M. bejeranoi*, which does not colonize an immune-privileged organ, immunomodulation is an effective survival strategy. Our study is the first to document the implementation of such a strategy in a *Myxobolus* species. The ability of the parasite to transmit an effective shutdown signal from a confined granulomata in the gills to the immune organs is intriguing.

## Data Availability

The data supporting the findings of this study are presented in the main text and its additional files. The raw sequence data were deposited in the NCBI SRA database under Bioproject accession PRJNA843447.

## Supplementary Materials

The following supporting information can be downloaded at: https://www.mdpi.com/article/10.3390/microorganisms10101893/s1, Table S1: Primers used in this study for PCR and RT-PCR; Table S2: Details of enriched GO/KEGG pathways supplementary to Figure 3; Table S3: Details on labels of enriched GO/KEGG pathways of Figure 4 networks; Table S4: Key secreted signaling / effectors significantly differentially expressed genes; Table S5: Details on labels of enriched GO/KEGG pathways of Figure 5 network; Table S6: Details on labels of significantly differentially expressed immune-related genes of Figure 6 network.
